# Breaking the Cycle, Cholesterol Cycling, and Synapse Damage in Response to Amyloid-β

**DOI:** 10.1177/1179069517733096

**Published:** 2017-12-06

**Authors:** Clive Bate

**Affiliations:** Department of Pathology and Pathogen Biology, The Royal Veterinary College, Hatfield,UK

**Keywords:** Cholesterol, amyloid, cholesterol ester, phospholipase A2, synapse

## Abstract

Soluble amyloid-β (Aβ) oligomers, a key driver of pathogenesis in Alzheimer disease, bind to cellular prion proteins (PrP^C^) expressed on synaptosomes resulting in increased cholesterol concentrations, movement of cytoplasmic phospholipase A_2_ (cPLA_2_) to lipid rafts and activation of cPLA_2_. The formation of Aβ-PrP^C^-cPLA_2_ complexes was controlled by the cholesterol ester cycle. Thus, Aβ activated cholesterol ester hydrolases which released cholesterol from stores of cholesterol esters; the increased cholesterol concentrations stabilised Aβ-PrP^C^-cPLA_2_ complexes. Conversely, cholesterol esterification reduced cholesterol concentrations causing the dispersal of Aβ-PrP^C^-cPLA_2_. In cultured neurons, the cholesterol ester cycle regulated Aβ-induced synapse damage; inhibition of cholesterol ester hydrolases protected neurons, whereas inhibition of cholesterol esterification increased the Aβ-induced synapse damage. Here, I speculate that a failure to deactivate signalling pathways can lead to pathology. Consequently, the esterification of cholesterol is a key factor in the dispersal of Aβ-induced signalling platforms and synapse degeneration.

## Commentary

A recent paper from my laboratory highlighted the involvement of the cholesterol ester cycle in response to amyloid-β (Aβ) oligomers, a key driver of pathogenesis in Alzheimer disease (AD).^1^ The study concentrated on events occurring at 2 levels. At a cellular level, we demonstrated that manipulation of the cholesterol ester cycle affected Aβ-induced synapse damage. The biochemistry and cell signalling associated with Aβ was also examined in isolated synaptosomes. To our knowledge, this was the first study to show that changes in membrane cholesterol concentrations mediated by the cholesterol ester cycle significantly affect a major cell signalling pathway.

Because cholesterol is such a key molecule involved in the regulation of membrane structure and function, it is not surprising that disturbances in cholesterol homeostasis are associated with neurodegenerative diseases^2^ and more specifically with the pathogenesis of AD, as reviewed by Chang et al.^3^ The amyloid hypothesis, the prevailing theory explaining the pathogenesis of AD, states that the accumulation of Aβ peptides within the brain is responsible.^4^ Although several studies have reported that cholesterol or cholesterol-binding proteins affect Aβ production, our study examined the role of cholesterol on the toxic effects of Aβ; primarily its effects on synapse degeneration as the loss of synapses and synaptic proteins shows a close correlation with the severity of dementia.^5^

The initial and key observation was that the addition of Aβ increased synaptic cholesterol concentrations, an observation that is consistent with reports of increased cholesterol concentrations in Aβ-positive synapses in the cortex of patients with AD.^6^ Surprisingly, this was not due to cholesterol synthesis, rather the Aβ-induced increase in synaptic cholesterol concentrations was controlled by the cholesterol ester cycle; it was accompanied by a corresponding reduction in cholesterol ester concentrations indicating the activation of a cholesterol ester hydrolase (CEH). Furthermore, selective CEH inhibitors blocked the Aβ-induced increase in synaptic cholesterol concentrations.

Cholesterol is highly enriched in synaptic membranes, and given that cholesterol concentrations in cell membranes are critical for the formation of signalling platforms in lipid rafts,^7^ we argued that fluctuations in cholesterol concentrations could alter the functions of lipid rafts. Lipid raft formation is associated with the aggregation of the cellular prion protein (PrP^C^), identified as a receptor for Aβ,^8^ by Aβ oligomers.^9^ Notably, the increase in synaptic cholesterol concentrations was associated with the toxic Aβ oligomers^10,11^ rather than non-toxic Aβ monomers.^12^ Here, we speculate that Aβ oligomers, but not monomers have the ability to cross-link cellular receptors, a hypothesis consistent with observations that synaptic abnormalities are caused by the cross-linkage of PrP^C^ with monoclonal antibodies.^13^ Cellular prion protein acts as a scaffold protein that organises signalling complexes and in neurons the clustering of specific glycosylphosphatidylinositols attached to PrP^C^ caused aberrant cell signalling and synapse degeneration.^14^

Cellular prion protein is associated with numerous cell signalling pathways including cytoplasmic phospholipase A_2_ (cPLA_2_)^15^ which leads to the production of platelet-activating factor (PAF) and prostaglandins. The observations that concentrations of prostaglandin E_2_ (PGE_2_) and PAF are raised in the brains of patients with AD when compared with non-demented controls^16,17^ suggest that aberrant activation of cPLA_2_ is associated with synapse degeneration and clinical symptoms.

**Figure 1.**
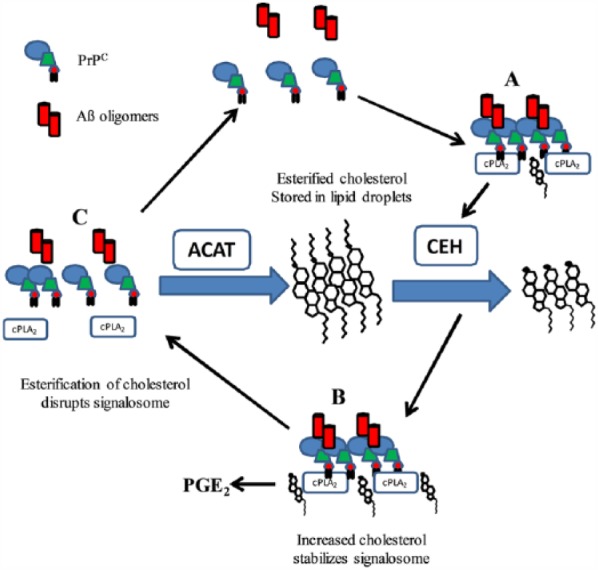
Putative control of synaptic signalosome created by Aβ oligomers: The binding of Aβ oligomers cross-links PrP^C^ within lipid rafts (A) recruits and activates cPLA_2._ Subsequently, CEHs are activated causing the release of cholesterol into the membrane; (B) the increase in cholesterol stabilises signalling complexes. cPLA_2_ is activated within the signalling platform releasing PGE_2_ which at high concentrations causes synapse degeneration. (C) ACAT activity resulted in the esterification of cholesterol, reduced free cholesterol concentrations in the membrane, and caused the dispersal of lipid raft signalling complexes. Aβ indicates amyloid-β; ACAT, acetyl-coenzyme A acetyltransferase; cPLA_2_, cytoplasmic phospholipase A_2_; PGE_2_, prostaglandin E_2_; PrP^C^, cellular prion proteins.

We hypothesised that Aβ oligomers cross-linked PrP^C^ leading to the activation of CEHs and increased cholesterol concentrations; these stabilise a signalling platform that included activated cPLA_2_ and led to increased production of PGE_2_ (Figure 1). This hypothesis was supported by the close correlations between the concentrations of cholesterol, raft-resident cPLA_2_, activated cPLA_2_, and PGE_2_ production following the addition of Aβ. Furthermore, pre-treatment with CEH inhibitors prevented the formation of Aβ-PrP^C^ complexes, the Aβ-induced increase in cholesterol, the movement of cPLA_2_ to lipid rafts, the activation of cPLA_2_, and the production of PGE_2_. When tested on cultured neurons, CEH inhibitors reduced the Aβ-induced synapse damage indicating that these enzymes are critical for Aβ toxicity. This suggests that the events measured within synaptosomes relate to the process of Aβ-induced synapse degeneration.

Time course studies demonstrated that the Aβ-induced increase in cholesterol/reduction in cholesterol esters, PrP^C^-Aβ complexes, and the amounts of cPLA_2_ within lipid rafts were all transient. Notably, the return of cholesterol/cholesterol ester concentrations to basal levels following esterification was closely associated with the dissociation of Aβ-PrP^C^ complexes and the return of cPLA_2_ to the cytoplasm. In addition, pre-treatment with selective inhibitors of acetyl-coenzyme A acetyltransferase (ACAT), an enzyme that esterifies cholesterol, resulted in increased Aβ-PrP^C^ complexes, higher cholesterol concentrations, increased time that cPLA_2_ spent within lipid rafts, increased activation of cPLA_2_, and higher PGE_2_ concentrations.

Conditions in which signalling platforms fail to dissociate may lead to sustained activation of signalling pathways and lead to cell disruption and disease. Consequently, the dissociation of signalling platforms is thought to be an important physiological process that limits the intensity of cell signalling. An altered cholesterol ester cycle resulting in accumulation of cholesterol esters has been reported in patients with AD.^18^ In this Commentary, we speculate that high concentrations of Aβ could ‘break the cycle’ by reducing the esterification of cholesterol and consequently preventing the dissociation of signalling platforms. In this respect, it was noteworthy that inhibition of ACAT in neuronal cultures significantly increased the Aβ-induced synapse damage. Acetyl-coenzyme A acetyltransferases may affect different aspects of AD pathogenesis. For example, ACAT inhibitors have been proposed as treatments for AD because they reduced Aβ production in studies where they were used throughout the course of an experimental disease.^19^ However, this study shows that ACAT inhibitors can increase synapse damage in the presence of Aβ. Consequently, ACAT inhibitors might be able to prevent the development of AD but maybe contraindicated in the latter stages of AD where concentrations of Aβ are already raised.

In summary, our article demonstrated the role of the cholesterol ester cycle in Aβ-induced cell signalling at synapses and synapse degeneration. The release of cholesterol stabilises the complexes formed between PrP^C^ and Aβ that activate cPLA_2_. Conversely, the esterification of cholesterol facilitates the dissociation of PrP^C^-Aβ complexes and deactivation of cPLA_2_.
